# Association between time in range of relative normoglycemia and in-hospital mortality in critically ill patients: a single-center retrospective study

**DOI:** 10.1038/s41598-022-15795-2

**Published:** 2022-07-13

**Authors:** Tomoya Okazaki, Akihiko Inoue, Takuya Taira, Shun Nakagawa, Kenya Kawakita, Yasuhiro Kuroda

**Affiliations:** 1 Emergency Medical Center, Kagawa University Hospital, 1750-1 Ikenobe, Miki, Kita, Kagawa 761-0793 Japan; 2 Department of Emergency and Critical Care Medicine, Hyogo Emergency Medical Center, 1-3-1 Wakinohamakaigandori, Chuo-ku, Kobe, Hyogo 651-0073 Japan; 3 Neurointensive Care Unit, Department of Neurosurgery, and Stroke and Epilepsy Center, TMG Asaka Medical Center, 1-1340 Mizonuma, Asaka, Saitama 351-8551 Japan

**Keywords:** Endocrine system and metabolic diseases, Diabetes

## Abstract

The aim of this single-center retrospective study was to investigate the association between the time in range (TIR) of relative normoglycemia (RN) and in-hospital mortality. We defined RN as measured blood glucose in the range of 70–140% of A1C-derived average glucose and absolute normoglycemia (AN) as 70–140 mg/dL. We conducted multivariate logistic regression analyses to examine the association between TIR of RN > 80% or TIR of AN > 80% up to 72 h after ICU admission and in-hospital mortality (Model 1 and Model 2, respectively). The discrimination of the models was assessed using the area under the receiver operating characteristic curve (AUROC). Among 328 patients, 35 died in hospital (11%). Model 1 showed that TIR of RN > 80% was associated with reduced in-hospital mortality (adjusted OR 0.16; 95% CI 0.06–0.43; P < 0. 001); however, Model 2 showed that the TIR of AN > 80% was not. The AUROC of Model 1 was significantly higher than that of Model 2 (0.84 [95% CI 0.77–0.90] vs. 0.79 [0.70–0.87], P = 0.008).Our findings provide a foundation for further studies exploring individualized glycemic management in ICUs.

## Introduction

Glycemic control is a key element in critically ill patients. The three domains of hyperglycemia, hypoglycemia, and glycemic variability are independently associated with mortality^1–3^. In addition, previous studies have found that pre-morbid glycemic status before intensive care unit (ICU) admission modulates the association between the three domains and mortality^4–6^. To consider pre-morbid glycemic conditions, an individualized approach using hemoglobin A1C-derived average glucose (ADAG), calculated using the equation 28.7 × A1C—46.7 mg/dL^7^, is being introduced^8–10^. A prospective study found that admission glucose level divided by ADAG was independently associated with in-hospital mortality in critically ill patients, whereas absolute glucose levels were not^9^.

In addition to the three domains, the time in the targeted blood glucose range is recognized as an essential factor. Previous studies have shown that a time in the targeted blood glucose range between 70 and 140 (or 180) mg/dL greater than 80% was associated with decreased mortality and other favorable outcomes^11–14^. Furthermore, the association between the time in the targeted blood glucose range and mortality was also reported to be dependent on pre-morbid glycemic status, indicating that the positive effects of a longer time in the targeted blood glucose range on mortality were not found in critically ill patients with poor antecedent glycemic control^11,13,14^. These findings suggest that the optimal blood glucose range needs to be tailored to each patient and provide a rationale for investigating the time in the targeted blood glucose range according to the antecedent glycemic control of individual patients.

This study investigated the association between the time in range (TIR) of relative normoglycemia, defined as measured blood glucose in the range of 70–140% of ADAG, and in-hospital mortality in critically ill patients who required emergency hospitalization.

## Results

A total of 600 patients were admitted to the ICU during the study period. Of these, 328 patients met the eligibility criteria for this study (Supplementary Fig. 1). Tables 1 and 2 show the patients’ baseline characteristics, glycemic profiles, and outcomes. Thirty-five patients (11%) died during hospitalization, 10 of whom were discharged from the ICU within 72 h. Between before and after the introduction of the new insulin protocol, there were significant differences in body mass index, mechanical ventilation, the TIR of relative normoglycemia, and time above 140% of ADAG (Supplementary Table 3).

**Table 1:** Basic characteristics: TIR of relative normoglycemia ≤ 80% vs. > 80%.

Characteristics	Overall N = 328	TIR ≤ 80% N = 156	TIR > 80% N = 172	*P* value
**ICU admission date**				0.19
Before November 1, 2020	137 (42)	71 (46)	66 (38)	
After November 1, 2020	191 (58)	85 (54)	106 (62)	
Age, years	72 (61, 79)	75 (66, 81)	70 (58, 77)	< 0.001
Female sex	112 (34)	47 (30)	65 (38)	0.14
Body mass index, kg/m^2^	22.2 (20.1, 24.8)	22.2 (20.0, 24.3)	22.6 (20.4, 25.2)	0.095
**ICU admission category**				0.51
Non-scheduled surgery	42 (13)	18 (12)	24 (14)	
Medical	286 (87)	138 (88)	148 (86)	
**Disease category**				0.015
Cardiovascular disorder	53 (16)	32 (21)	21 (12)	
Respiratory disorder	27 (8.2)	19 (12)	8 (4.7)	
Gastrointestinal disorder	41 (12)	19 (12)	22 (13)	
Neurologic disorder	107 (33)	40 (26)	67 (39)	
Trauma	58 (18)	25 (16)	33 (19)	
Others	42 (13)	21 (13)	21 (12)	
Sepsis	37 (11)	17 (11)	20 (12)	0.83
APACHE II score	18 (14, 24)	20 (15, 26)	17 (13, 22)	< 0.001
SOFA score	6 (4, 8)	7 (4, 9)	5 (3, 7)	< 0.001
Charlson comorbidity index score	1 (0, 3)	1 (0, 3)	1 (0, 2)	< 0.001
A1C, %	5.80(5.5, 6.5)	6.1 (5.5, 6.9)	5.7 (5.4, 6.2)	< 0.001
ADAG ^a^, mg/dL	120 (111, 140)	127 (111, 151)	117 (108, 131)	< 0.001
Diagnosed diabetes	98 (30)	68 (44)	30 (17)	< 0.001
**Management in ICU**				
Vasopressor use	88 (27)	46 (29)	42 (24)	0.30
Mechanical ventilation	153 (47)	75 (48)	78 (45)	0.62
Renal replacement therapy	22 (6.7)	9 (5.8)	13 (7.6)	0.52

Data are expressed as median (interquartile range) or number (%).

*TIR* time in range, *APACHE* Acute Physiology and Chronic Health Evaluation, *SOFA* Sequential Organ Failure Assessment, *ADAG* A1C-derived average glucose.

^a^A1C-derived average glucose (mg/dL) can be obtained from the following formula: 28.7 × A1C (%) − 46.7.

**Table 2:** Glycemic profile and outcomes: TIR of relative normoglycemia ≤ 80% vs. > 80%

Variables	Overall N = 328	TIR ≤ 80 N = 156	TIR > 80 N = 172	*P* value
**Glycemic profile**^**a**^				
The number of blood glucose measurements	14 (11, 17)	15 (12, 19)	13 (11, 16)	< 0.001
Mean blood glucose, mg/dL	130 (114, 158)	151 (130, 187)	121 (110, 132)	< 0.001
Coefficient of variation, %	19 (14, 27)	26 (19, 33)	15 (11, 20)	< 0.001
Hypoglycemia (< 70 mg/dL)	24 (7.3)	15 (9.6)	9 (5.2)	0.13
Moderate hypoglycemia (≥ 40 mg/dL, < 70 mg/dL)	20 (6.1)	12 (7.7)	8 (4.7)	0.25
Severe hypoglycemia (< 40 mg/dL)	4 (1.2)	3 (1.9)	1 (0.6)	0.35
TIR of absolute normoglycemia^b^, %	70 (33, 91)	50 (17, 69)	86 (67, 100)	< 0.001
TIR of absolute normoglycemia^b^ > 80%	123 (38)	20 (13)	103 (60)	< 0.001
Time below 70 mg/dL, %	0 (0, 0)	0 (0, 0)	0 (0, 0)	0.11
Time above 140 mg/dL, %	29 (8, 63)	50 (29, 81)	13 (0, 33)	< 0.001
TIR of relative normoglycemia^c^, %	83 (65, 100)	62 (50, 73)	96 (89, 100)	< 0.001
Time below 70% of ADAG^d^, %	0.0 (0.0, 9)	0.0 (0.0, 27)	0.0 (0.0, 0.0)	< 0.001
Time above 140% of ADAG^d^, %	7 (0.0, 25)	25 (0.0, 40)	0 (0.0, 7)	< 0.001
**Outcomes**				
ICU length of stay, days	4.7 (2.8, 8.4)	5.6 (2.7, 8.2)	4.4 (2.8, 8.6)	0.73
ICU mortality	20 (6.1)	17 (11)	3 (1.7)	< 0.001
Hospital length of stay, days	20 (13, 33)	19 (12, 33)	21 (13, 34)	0.28
Hospital mortality	35 (11)	30 (19)	5 (2.9)	< 0.001

Data are expressed as median (interquartile range) or number (%).

*TIR* time in range, *ADAG* A1C-derived average glucose.

^a^During the first 72 h after ICU admission or ICU stay, whichever shorter.

^b^Absolute normoglycemia was defined as measured blood glucose levels in the range of 70 to 140 mg/dL.

^c^Relative normoglycemia was defined as measured blood glucose levels in the range of 70 to 140 of ADAG.

^d^ 1C-derived average glucose (mg/dL) can be obtained from the following formula: 28.7 × A1C (%) − 46.7.

Patients with a TIR of relative normoglycemia > 80% were younger and had different disease categories, lower disease severity, lower CCI, and lower A1C levels than those with a TIR of relative normoglycemia ≤ 80%. Regarding the glycemic profile, patients with a TIR of relative normoglycemia > 80% had lower mean blood glucose, lower glucose variability, and a longer TIR of absolute normoglycemia (Table 2). In addition, in-hospital mortality was significantly lower in the TIR of relative normoglycemia > 80% group. Supplementary Table 4 summarizes the baseline characteristics, glycemic profile, and outcomes according to the TIR of absolute normoglycemia. We observed some differences that were similar to the differences between the TIR of relative normoglycemia ≤ 80% and > 80%.

Table 3 and Supplementary Table 5 summarize the results of the multivariate logistic regression analyses. Although Model 1 showed that a TIR of relative normoglycemia > 80% was significantly associated with reduced in-hospital mortality (adjusted odds ratio [OR]: 0.16; 95% confidence interval [CI] 0.06–0.43; P < 0. 001), Model 2 showed that a TIR of absolute normoglycemia > 80% was not. The AUROC of Model 1 was significantly higher than that of Model 2 (0.84 [95% CI 0.77–0.90] vs. 0.79 [0.70–0.87], P = 0.008).

**Table 3:** Multivariate logistic regression analyses for hospital mortality.

Model ^a^	Variable	Adjusted OR (95 CI)	*P* value	AUROC (95 CI)	*P* value^b^
1	TIR of relative normoglycemia > 80% (vs. ≤ 80%)	0.16 (0.06, 0.43)	< 0.001	0.84 (0.77, 0.90)	0.008
2	TIR of absolute normoglycemia > 80% (vs. ≤ 80%)	0.44 (0.15, 1.23)	0.118	0.79 (0.70, 0.87)	

*TIR* time in range, *OR* odds ratio, *AUROC* area under the receiver operating characteristic curve.

^a^Each model was adjusted according to age, sex, Acute Physiology and Chronic Health Evaluation II score and Charlson comorbidity index.

^b^*P* value for the comparison of the AUROC of the two models.

**Figure 1:**
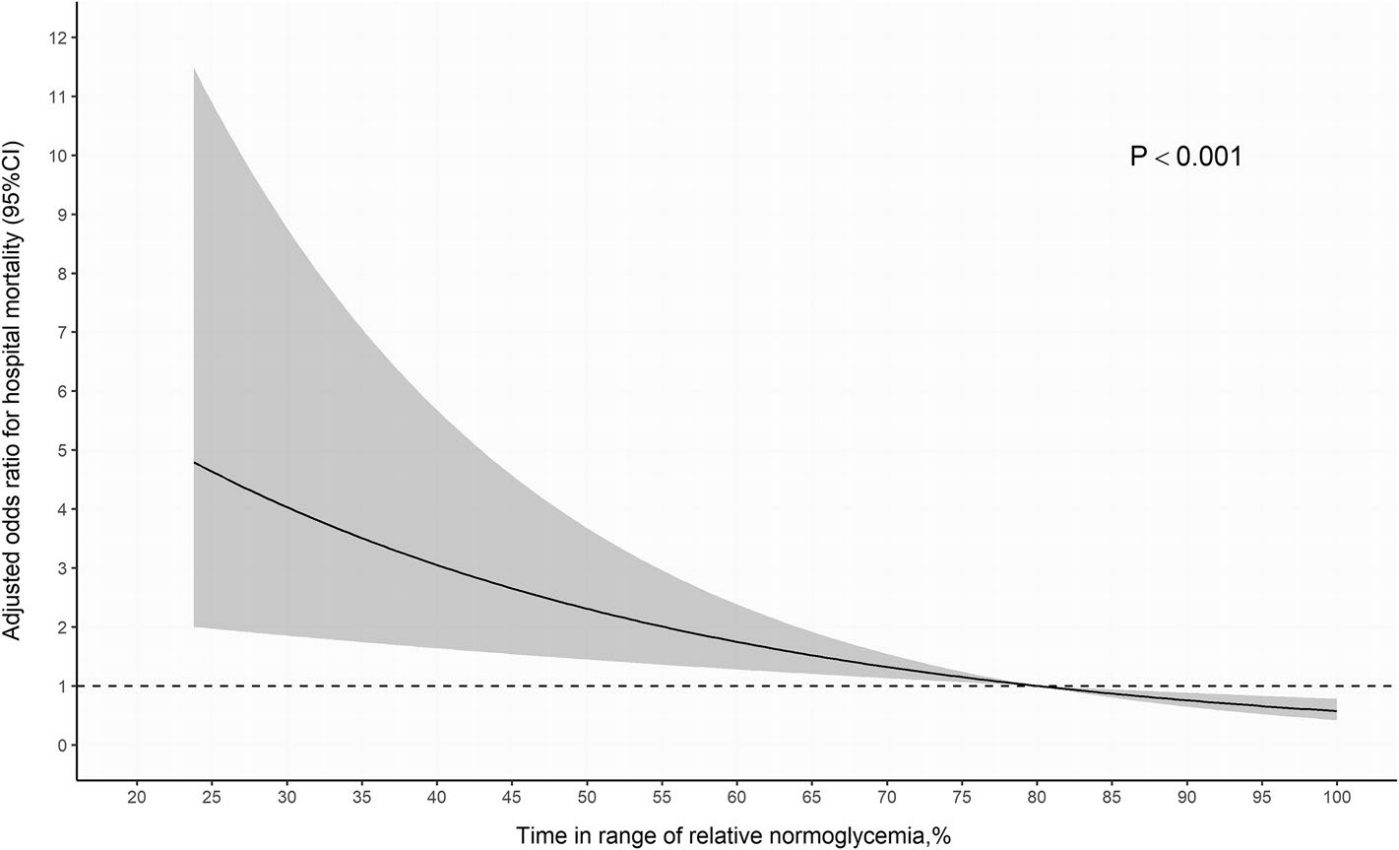
Adjusted odds ratio of the time in range of relative normoglycemia for in-hospital mortality according to the logistic regression model. *CI* confidence interval.

Figure 1 shows the relationship between the TIR of relative normoglycemia as a continuous variable and in-hospital mortality calculated using multivariate logistic regression. The adjusted OR of in-hospital mortality increased significantly with a shorter TIR of relative normoglycemia; however, this relationship was not observed for the TIR of absolute normoglycemia (Supplementary Fig. 2).

**Figure 2:**
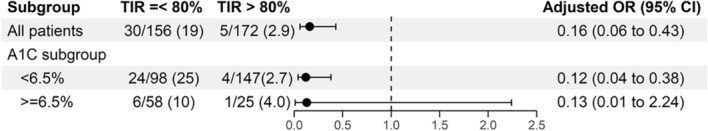
Adjusted odds ratios of the time in range of relative normoglycemia > 80% for in-hospital mortality according to A1C levels. *TIR* time in range, *OR* odds ratio, *CI* confidence interval.

Subgroup analyses according to A1C are shown in Fig. 2 and Supplementary Table 6. Although we observed an association between a TIR of relative normoglycemia > 80% and reduced mortality in patients with A1C < 6.5%, we did not find an association in those with A1C ≥ 6.5%. However, no significant interactions regarding A1C levels were found (adjusted OR: 2.11; 95% CI: 0.17–26.57; P = 0.562). On the other hand, the association between a TIR of absolute normoglycemia > 80% and mortality was almost significantly dependent on A1C levels.

Supplementary Table 7 summarizes the result of subgroup analyses according to blood glucose measurement interval. The median measurement interval was 4.7 h and patients were divided into two groups based on this value. The association between a TIR of relative normoglycemia > 80% and reduced in-hospital mortality was observed in both groups.

Supplementary Table 8 shows the time-varying Cox regression analysis result for 28-day mortality. As in the main analysis, a TIR of relative normoglycemia > 80% was significantly associated with reduced 28-day mortality (adjusted hazard ratio: 0.21; 95% CI: 0.08–0.58; P = 0. 002).

## Discussion

This single-center retrospective study found that a shorter TIR of relative normoglycemia was significantly associated with higher in-hospital and 28-day mortality, whereas the TIR of absolute normoglycemia was not. Additionally, the association between the TIR of relative normoglycemia and in-hospital mortality did not depend on A1C levels (and blood glucose measurement interval); however, the TIR of absolute normoglycemia was almost significantly dependent on A1C levels.

One of our findings was that the TIR of absolute normoglycemia (70–140 mg/dL) was significantly associated with in-hospital mortality only among patients with A1C < 6.5% but not among those with A1C ≥ 6.5%, which was consistent with previous studies investigating a time in targeted blood glucose range of 70–139 mg/dL^13^ or a time in blood glucose range using the more liberal upper limit (180 mg/dL)^14^. Therefore, our results reaffirm that the range of 70–140 mg/dL cannot be a target for all patients, as well as the importance of individual blood glucose targets in accordance with A1C levels.

We demonstrated that the TIR of relative normoglycemia, defined as 70–140% of ADAG, was significantly associated with in-hospital mortality among all patients; however, the TIR of absolute normoglycemia was not. Similar to our study, several studies have suggested that relative glycemic metrics guided by ADAG may be better than absolute glycemic metrics. A secondary analysis of a randomized control trial involving 192 patients with acute myocardial infarction showed that the mean blood glucose level during the first 24 h divided by ADAG was associated with composite outcomes, including death, but absolute glucose level was not^17^. A prospective study found that admission glucose level divided by ADAG was independently associated with in-hospital mortality in critically ill patients, whereas absolute glucose levels were not^9^.

Adapting our definition of relative normoglycemia to patients with a higher A1C implies two points. The first point is permitting hyperglycemia. A retrospective observational study revealed lower in-hospital mortality with a higher time-weighted average blood glucose level during ICU stay in patients with diabetes with higher A1C levels^18^. Another retrospective study involving over 5000 patients found that an increased mean blood glucose level was associated with decreased mortality in patients with A1C ≥ 8.0%, which was precisely opposite to the relationship in patients with A1C < 6.5%^6^. The second point is setting a hypoglycemic threshold higher than 70 mg/dL in patients with high A1C levels. A retrospective observational study found a stronger association between absolute hypoglycemia and mortality in patients with higher A1C than in those with lower A1C levels^5^. Additionally, relative hypoglycemia, defined as < 70% of ADAG but not below 70 mg/dL, was reported to be a mortality risk factor^16^. On the other hand, because the range considered as relative normoglycemia under our definition widens with higher A1C, there is concern about glycemic variability which is associated with mortality^3,19,20^. Although previous studies reported that higher A1C levels attenuated the relationship between glycemic variability and mortality, further studies are needed.

In contrast, compared with setting a higher glycemic target for patients with higher A1C levels, there is less evidence for imposing a lower glycemic target in patients with lower A1C levels. Generally, targeting lower glucose levels is more likely to cause mortality-related hypoglycemia^2,15,21^. On the other hand, it remains possible that lower glucose control with minimal hypoglycemia may be more effective than conventional control^22^, and the present study might support this hypothesis. Furthermore, under our definition of relative normoglycemia, the lower threshold of relative normoglycemia was lower than 70 mg/dL for patients with A1C < 5.1%. A post-hoc analysis of a randomized control trial showed a trend towards improved outcomes with lower target glucose control compared with conventional control in patients with A1C < 5.0%, even though hypoglycemia was more prevalent (adjusted hazard ratio 0.60, 95% CI 0.35–1.01; P = 0.052). However, only 19 patients had A1C less than 5.1%, and 17 of them were not below 70 mg/dL in our study. Therefore, it is unclear whether it is safe to set the lower limit of relative normoglycemia to less than 70 mg/dL, and additional studies are warranted.

The primary significance of our findings is that the TIR of relative normoglycemia may be better than that of absolute normoglycemia for predicting in-hospital mortality, emphasizing the importance of A1C-derived individualized glycemic management. Although a randomized control trial did not demonstrate the superiority of individualized glycemic management using ADAG compared with conventional management using a target of ≤ 180 mg/dL^21^, it took time to obtain A1C; therefore, the TIR using ADAG may have been insufficient. Thus, a controlled trial with an environment where A1C is rapidly available and an appropriate blood glucose management protocol is necessary to precisely evaluate A1C-derived individualized glycemic management.

The strength of the present study is that this is the first report of an association between a TIR of 70–140% of ADAG and in-hospital mortality, which suggests the possibility of individualized glycemic management targeting ADAG in the ICU.

However, our study has several limitations. First, this was a single-center, retrospective study. Therefore, although we performed multivariate logistic regression analyses, selection biases and uncontrolled confounding factors could have existed. Furthermore, we could not mention a causal relationship because of the nature of observational studies. Second, we could not evaluate the interactions between the TIR of relative normoglycemia and other glycemic metrics. Third, we did not consider the presence or absence of diabetes. However, previous studies have shown that A1C has a stronger effect on the relationship between glucose measurements and outcomes than diabetes^13,14^. Fourth, we defined relative normoglycemia as measured blood glucose levels in the range of 70–140% of ADAG based on previous studies; however, the external validity of this range has not yet been assessed. Moreover, we did not evaluate long-term outcomes. Finally, the current study was carried out only at Kagawa University Hospital; therefore, it is unknown whether our findings can be extrapolated to other institutions and regions.

## Methods

### Study design and setting

This single-center retrospective observational study was conducted at the Emergency Medical Center of Kagawa University Hospital, a 613-bed academic teaching institution in Japan, between January 1, 2020, and July 31, 2021. The Emergency Medical Center has a 10-bed ICU with a patient-nurse ratio of 2:1 and a 12-bed intermediate care unit with a patient-nurse ratio of 4:1. Kagawa University Hospital has another ICU that treats patients after scheduled surgeries and those from general wards. Therefore, we treated only patients requiring emergency admission to the ICU from an emergency department, an outpatient department, and other hospitals. This study was reviewed and approved by the Institutional Review Board of Kagawa University Hospital (2021-132). The committee waived the requirement for patient consent due to the retrospective nature of the study.

### Participants

We included all ICU admission data obtained from patients aged ≥ 18 years. Patients with coronavirus disease 2019 were treated in other wards. Therefore, these patients were not included in the study. We excluded ICU admission data that met any of the following criteria: (1) the second and subsequent ICU admissions of patients with multiple ICU admissions during the study period; (2) patients with diabetic ketoacidosis or hyperosmolar hyperglycemic state; (3) ICU stay of less than 24 h; (4) patients without A1C values; or (5) patients with less than five blood glucose measurements during the first 72 h after ICU admission or ICU stay, whichever was shorter.

### Data collection

The following baseline data were collected: admission date, age, sex, body mass index, admission category, disease category, Acute Physiology Chronic and Health Evaluation (APACHE) II score, Sequential Organ Failure Assessment score within 24 h of ICU admission, A1C on admission or within the previous 3 months, and Charlson comorbidity index (CCI). In addition, we recorded the following management data in the ICU: blood glucose readings during the first 72 h after ICU admission (or ICU stay whichever shorter), vasopressor use, mechanical ventilation, renal replacement therapy, and tracheostomy. We also collected data related to the following outcomes: length of mechanical ventilation, length of ICU stay, ICU mortality, length of hospital stay, and in-hospital mortality.

### Glycemic management in the ICU

We used glucose-free infusion and increased nutrition daily according to our protocol. We routinely obtained arterial blood gas at ICU admission and every 6 h during the ICU stay. Blood glucose levels were measured using a blood gas analyzer. We began using a new insulin infusion protocol on November 1, 2020. Before the introduction, we started continuous insulin infusion when the blood glucose level reached ≥ 200 mg/dL and adjusted as necessary with reference to the NICE-SUGAR study^15^. After the new insulin infusion protocol, the glucose target value depended on A1C. For patients with an A1C level of less than 8.0%, we started continuous insulin infusion when the blood glucose level exceeded 180 mg/dL and adjusted the insulin dosage to a target of 140–180 mg/dL. On the other hand, if the A1C level was above 8.0%, continuous insulin infusion was started when the blood glucose level exceeded 220 mg/dL and adjusted to a target of 180–220 mg/dL. In both cases, once we started insulin infusion, enteral nutrition was switched to continuous administration, and blood glucose monitoring was performed every 2 h. The protocol details are shown in Supplementary Table 1a,b. If we did not obtain A1C on ICU admission, we managed the patients using the insulin protocol for an A1C level of less than 8.0%.

### Definitions

We used A1C to calculate the ADAG of each patient: ADAG (mg/dL) = 28.7 × A1C (%)—46.7^7^. The TIR of relative normoglycemia was defined as the time spent between 70 and 140% of the ADAG during the first 72 h (or ICU stay, whichever was shorter) divided by 72 h (or ICU stay, whichever was shorter). The lower threshold was adopted from a previous study on relative hypoglycemia^16^ based on an average blood glucose level of 100 mg/dL in healthy people and a hypoglycemic threshold of 70 mg/dL. In contrast, 140 mg/dL is widely accepted as the threshold for hyperglycemia. Therefore, we defined the upper limit of relative normoglycemia as a 40% increase from the ADAG. Supplementary Table 2 shows some examples of the relative normoglycemic ranges according to A1C levels. We defined the TIR of absolute normoglycemia as the time spent between 70 and 140 mg/dL during the first 72 h (or ICU stay, whichever was shorter) divided by 72 h (or ICU stay, whichever was shorter). To explore the importance of relative and absolute normoglycemia in the early phase, we focused only on the first 72 h.

### Exposure and endpoint

The primary exposure was the TIR of relative normoglycemia > 80% during the first 72 h after ICU admission (or ICU stay, whichever was shorter). We adopted this 80% threshold based on previous studies^11–14^. The secondary exposure was the TIR of absolute normoglycemia > 80% during the first 72 h after ICU admission (or ICU stay, whichever was shorter). The primary endpoint was in-hospital mortality.

### Statistical analysis

Continuous variables are expressed as medians with interquartile ranges and were compared using the Mann–Whitney U test. Categorical variables are expressed as numbers with percentages and were compared using chi-square test or Fisher’s exact test.

We divided the patients into two groups according to the primary or secondary exposure and compared baseline characteristics, management in the ICU, glycemic profile, and outcomes. We conducted multivariate logistic regression analyses adjusted for age, sex, APACHE II score, and CCI to examine the association between the TIR of relative normoglycemia > 80% or the TIR of absolute normoglycemia > 80% and in-hospital mortality (Model 1 and Model 2, respectively). These factors were chosen a priori based on clinical persuasion and previous studies^13,14^. The discrimination of the models was assessed using the area under the receiver operating characteristic curve (AUROC).

We performed two pre-defined sensitivity analyses. First, to assess the relationship between the TIR of relative or absolute normoglycemia as a continuous variable and in-hospital mortality, we conducted a logistic regression analysis controlling for the same confounders in the primary analysis (age, sex, APACHE II score, and CCI). Second, to evaluate the heterogeneity of different levels of A1C, we conducted a subgroup analysis of patients with an A1C of < 6.5% or ≥ 6.5%. Furthermore, we performed two additional sensitivity analyses. First, because the frequency of blood glucose measurement was different across patients, we conducted a subgroup analysis of patients with shorter or longer measurement intervals to determine whether the association between TIIR of relative normoglycemia and the outcome could depend on the frequency of BG measurements. We assigned patients into groups with shorter or longer measurement intervals according to the median measurement interval calculated by dividing 72 h (or ICU stay, whichever was shorter) by the number of blood glucose measurements. Second, to assess the robustness of our main analysis, we conducted a Cox regression analysis for 28-day mortality including the TIR of relative normoglycemia > 80 as a time-varying covariate, adjusted for age, sex, APACHE II score, and CCI.

A two-sided P*-*value < 0.05 was considered statistically significant. All statistical analyses were performed using R (version 4.1.1, R Foundation for Statistical Computing, Vienna, Austria).

## Supplementary Information

Supplementary Information.

## Data Availability

The datasets used in the current study are available from the corresponding author on reasonable request.
