# Supplementary material for: Association between time in range of relative normoglycemia and in-hospital mortality in critically ill patients: a single-center retrospective study
Source: Sci Rep. 2022 Jul 13;12:11864. doi: 10.1038/s41598-022-15795-2 (PMC9277973; doi:10.1038/s41598-022-15795-2)
Supplement: Supplementary file 1 — Supplementary Information. [file 41598_2022_15795_MOESM1_ESM.docx]

**Supplementary Table 1** The insulin protocol of Kagawa University Hospital

1. For patients with A1C ≤ 8.0%

| **Current BG**  **(mg/dl)** | **Difference between previous BG and current BG (mg/dl)** | **Management** | **When to recheck** |
| --- | --- | --- | --- |
| ~89 |  | Stop insulin infusion and IV dextrose 10mg | Recheck in 15 minutes and 1 hour |
| 90~109 |  | Stop insulin infusion | Recheck in 1 hour  Restart insulin at half dose  if the retest value is > 140 mg/dl  Then recheck in 1 hour |
| 110~139 | ≧ 40mg/dl | Stop insulin infusion | Recheck in 1 hour  Restart insulin at half dose  if the retest value is > 140 mg/dl  Then recheck in 2 hours |
|  | < 40mg/dl | Reduce insulin to 0.5 units/hour  or  Stop insulin infusion  if insulin dosage is less than 0.5 units/hour | Recheck in 2 hours  Restart insulin at half dose  if the retest value is > 140 mg/dl  Then recheck in 2 hours |
| 140~180 | > 80mg/dl | Stop insulin infusion | Recheck in 2 hours  Restart insulin at half dose  if the retest value is > 140 mg/dl  Then recheck in 2 hours |
|  | 40-80mg/dl | Reduce insulin infusion by half | Recheck in 2 hours |
|  | < 40mg | Keep insulin infusion dosage | Recheck in 2 hours or  4 hours if the previous value was also 140-180 mg/dl |
| 181~250 | > 80mg/dl | Reduce insulin infusion by half | Recheck in 2 hours |
|  | 40-80mg/dl | Keep insulin infusion dosage | Recheck in 2 hours |
|  | < 40mg | Increase insulin infusion by 1 unit/hour | Recheck in 2 hours |
| 251~ | ≧ 40mg/dl | Keep insulin infusion dosage | Recheck in 2 hours |
|  | < 40mg/dl | Insulin IV 1 unit and  increase insulin infusion by 1 unit/hour | Recheck in 2 hours |

1. For patients with A1C > 8.0%

| **Current BG**  **(mg/dl)** | **Difference between previous BG and current BG (mg/dl)** | **Management** | **When to recheck** |
| --- | --- | --- | --- |
| ~129 |  | Stop insulin infusion and IV dextrose 10mg | Recheck in 15 minutes and 1 hour |
| 130~149 |  | Stop insulin infusion | Recheck in 1 hour  Restart insulin at half dose  if the retest value is > 180 mg/dl  Then recheck in 1 hour |
| 150~179 | ≧ 40mg/dl | Stop insulin infusion | Recheck in 1 hour  Restart insulin at half dose  if the retest value is > 180 mg/dl  Then recheck in 2 hours |
|  | < 40mg/dl | Reduce insulin to 0.5 units/hour  or  Stop insulin infusion  if insulin dose is less than 0.5 units/hour | Recheck in 2 hours  Restart insulin at half dose  if the retest value is > 180 mg/dl  Then recheck in 2 hours |
| 180~220 | > 80mg/dl | Stop insulin infusion | Recheck in 2 hours  Restart insulin at half dose  if the retest value is > 180 mg/dl  Then recheck in 2 hours |
|  | 40-80mg/dl | Reduce insulin infusion by half | Recheck in 2 hours |
|  | < 40mg | Keep insulin infusion dosage | Recheck in 2 hours or  4 hours if the previous value was also 180-220 mg/dl |
| 221~300 | > 80mg/dl | Reduce insulin infusion by half | Recheck in 2 hours |
|  | 40-80mg/dl | Keep insulin infusion dosage | Recheck in 2 hours |
|  | < 40mg | Increase insulin infusion by 1 unit/hour | Recheck in 2 hours |
| 301~ | ≧ 40mg/dl | Keep insulin infusion dosage | Recheck in 2 hours |
|  | < 40mg/dl | Insulin IV 1 unit and  increase insulin infusion by 1 unit/hour | Recheck in 2 hours |

**Supplementary Table 2** The range of relative normoglycemia according to glycated hemoglobin A1c levels

|  |  | **The range of relative normoglycemia ^b^** | |
| --- | --- | --- | --- |
| **Glycated hemoglobin A1c (%)** | **A1C-derived average glucose (mg/dl) ^a^** | **Lower limit (mg/dl)** | **Upper limit (mg/dl)** |
| 4.0 | 68.1 | 47.7 | 95.3 |
| 4.5 | 82.5 | 57.7 | 115.4 |
| 5.0 | 96.8 | 67.8 | 135.5 |
| 5.1 | 100 | 70 | 140 |
| 5.5 | 111.2 | 77.8 | 155.6 |
| 6.0 | 125.5 | 87.9 | 175.7 |
| 6.5 | 139.9 | 97.9 | 195.8 |
| 7.0 | 154.2 | 107.9 | 215.9 |
| 7.5 | 168.6 | 118.0 | 236.0 |
| 8.0 | 182.9 | 128.0 | 256.1 |
| 8.5 | 197.3 | 138.1 | 276.2 |
| 9.0 | 211.6 | 148.1 | 296.2 |
| 9.5 | 226.0 | 158.2 | 316.3 |
| 10.0 | 240.3 | 168.2 | 336.4 |
| 10.5 | 254.7 | 178.3 | 356.5 |
| 11.0 | 269.0 | 188.3 | 376.6 |
| 11.5 | 283.4 | 198.3 | 396.7 |
| 12.0 | 297.7 | 208.4 | 416.8 |

^a^ A1C-derived average glucose (mg/dl) can be obtained from the following formula: 28.7×A1C -46.7

^b^ Relative normoglycemia was defined as measured blood glucose levels in the range of 70 to 140 of A1C-derived average glucose

**Supplementary Figure 1. Patient screening flow**


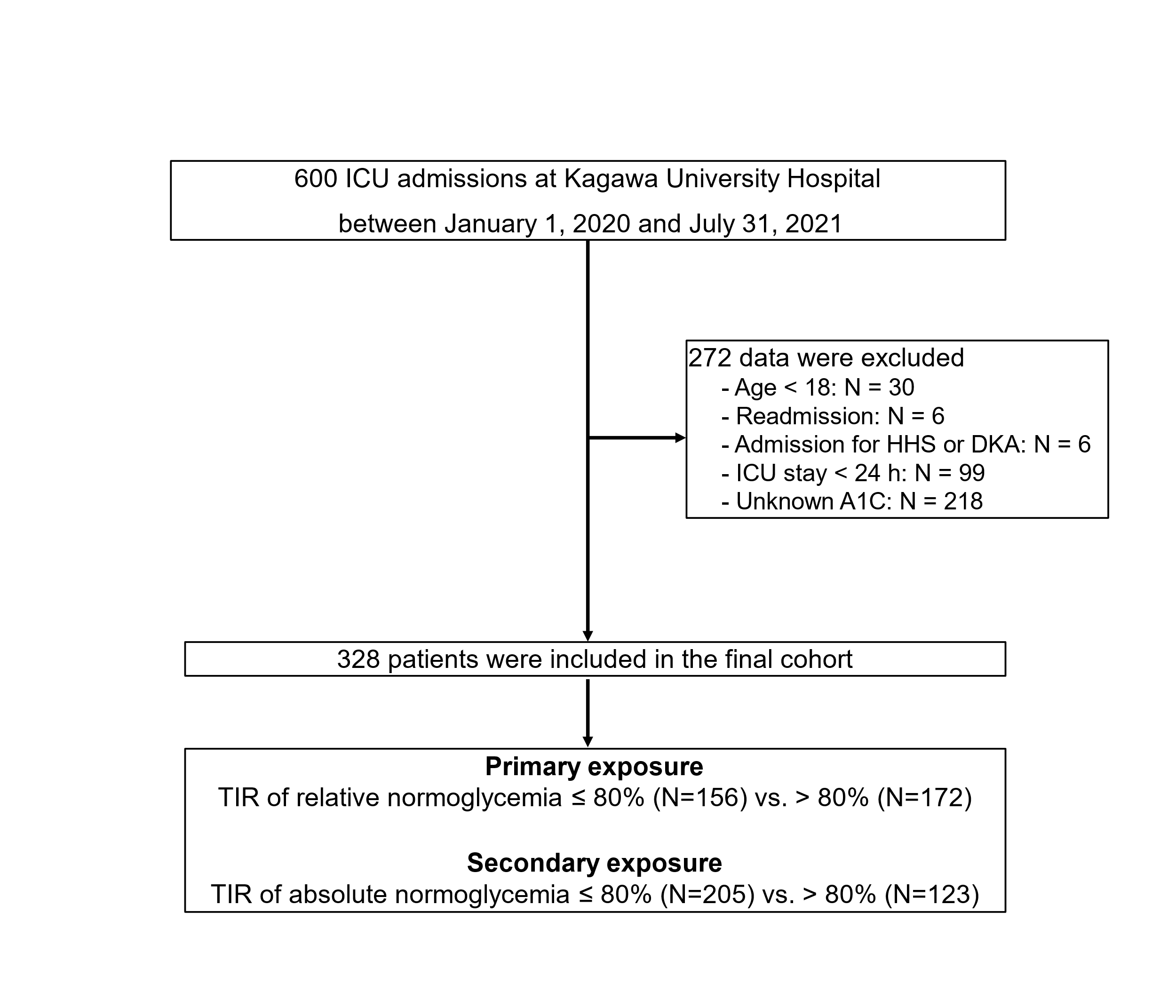


*TIR* time in range

Relative normoglycemia was defined as measured blood glucose levels in the range of 70 to 140 of A1C-derived average glucose.

Absolute normoglycemia was defined as measured blood glucose levels in the range of 70 to 140 mg/dl.

**Supplementary Table 3** Basic characteristics, glycemic profile and outcomes: Before vs. after the introduction of the new insulin protocol

| Variables |  | Before  N = 137 | After  N = 191 | *P* value |
| --- | --- | --- | --- | --- |
| Age, years |  | 72 (62, 82) | 72 (61, 78) | 0.30 |
| Female sex |  | 44 (32) | 68 (36) | 0.51 |
| Body mass index, kg/m^2^ |  | 21.6 (19.5, 24.0) | 23.1 (20.6, 25.2) | 0.01 |
| ICU admission category |  |  |  | 0.14 |
|  | Non-scheduled surgery | 22 (16) | 20 (10) |  |
|  | Medical | 115 (84) | 171 (90) |  |
| Disease category |  |  |  | 0.98 |
|  | Cardiovascular disorder | 24 (18) | 29 (15) |  |
|  | Respiratory disorder | 10 (7.3) | 17 (8.9) |  |
|  | Gastrointestinal disorder | 17 (12) | 24 (13) |  |
|  | Neurologic disorder | 46 (34) | 61 (32) |  |
|  | Trauma | 24 (18) | 34 (18) |  |
|  | Others | 16 (12) | 26 (14) |  |
| Sepsis |  | 21 (15) | 16 (8.4) | 0.050 |
| APACHE II score |  | 19 (15, 24) | 18 (13, 24) | 0.21 |
| SOFA score |  | 6.0 (4.0, 8.0) | 5.0 (3.0, 8.0) | 0.40 |
| Charlson comorbidity index score |  | 1.00 (0.00, 3.00) | 1.00 (0.00, 3.00) | 0.39 |
| A1C, % |  | 5.80 (5.50, 6.60) | 5.80 (5.50, 6.40) | 0.98 |
| ADAG ^a^, mg/dl |  | 120 (111, 143) | 120 (111, 137) | 0.98 |
| Diagnosed diabetes |  | 42 (31) | 56 (29) | 0.79 |
| **Management in ICU** |  |  |  |  |
| Vasopressor use |  | 33 (24) | 55 (29) | 0.34 |
| Mechanical ventilation |  | 73 (53) | 80 (42) | 0.041 |
| Renal replacement therapy |  | 7 (5.1) | 15 (7.9) | 0.33 |
| **Glycemic profile^b^** |  |  |  |  |
| The number of blood glucose measurements | | 14.0 (12.0, 18.0) | 14.0 (10.5, 17.0) | 0.53 |
| Mean blood glucose, mg/dl |  | 131 (114, 160) | 130 (114, 156) | 0.39 |
| Coefficient of variation, |  | 20 (15, 27) | 18 (13, 27) | 0.13 |
| Hypoglycemia (< 70 mg/dL) |  | 10 (7.3) | 14 (7.3) | 1.00 |
| Moderate hypoglycemia (≥ 40mg/dl, < 70mg/dl) | | 10 (7.3) | 10 (5.2) | 0.44 |
| Severe hypoglycemia (< 40mg/dl) |  | 0 (0) | 4 (2.1) | 0.14 |
| TIR of absolute normoglycemia^c^, % | | 67 (33, 90) | 71 (36, 91) | 0.46 |
| TIR of absolute normoglycemia^c^ > 80 % | | 46 (34) | 77 (40) | 0.21 |
| Time below 70 mg/dl, % |  | 0.00 (0.00, 0.00) | 0.00 (0.00, 0.00) | 0.96 |
| Time above 140 mg/dl, % |  | 33 (8, 67) | 29 (7, 62) | 0.32 |
| TIR of relative normoglycemia^d^, % | | 79 (62, 92) | 83 (67, 100) | 0.044 |
| TIR of relative normoglycemia^d^ > 80 % | | 66 (48) | 106 (55) | 0.19 |
| Time below 70 % of ADAG^a^, % |  | 0 (0, 12) | 0 (0, 8) | 0.38 |
| Time above 140 % of ADAG^a^, % |  | 8 (0, 25) | 0 (0, 23) | 0.021 |
| **Outcomes** |  |  |  |  |
| ICU length of stay, days |  | 5.5 (2.9, 9.2) | 4.1 (2.7, 7.2) | 0.059 |
| ICU mortality |  | 9 (6.6) | 11 (5.8) | 0.76 |
| Hospital length of stay, days |  | 21 (13, 32) | 20 (12, 33) | 0.73 |
| Hospital mortality |  | 10 (7.3) | 25 (13) | 0.094 |

Data are expressed as median (interquartile range) or number (%)

*TIR* time in range, *APACHE* Acute Physiology and Chronic Health Evaluation, *SOFA* Sequential Organ Failure Assessment, *ADAG* A1C derived average glucose

^a^ A1C-derived average glucose (mg/dl) can be obtained from the following formula: 28.7×A1C (%) -46.7

^b^ During the first 72 h after ICU admission or ICU stay, whichever shorter

^c^ Absolute normoglycemia was defined as measured blood glucose levels in the range of 70 to 140 mg/dl.

^d^ Relative normoglycemia was defined as measured blood glucose levels in the range of 70 to 140 of ADAG.

**Supplementary Table 4** Basic characteristics, glycemic profile and outcomes: TIR of absolute normoglycemia ≤ 80% vs. > 80%

| Variables |  | Overall,  N = 328 | TIR ≤ 80%  N = 205 | TIR > 80%  N = 123 | *P* value |
| --- | --- | --- | --- | --- | --- |
| **Basic characteristics** |  |  |  |  |  |
| ICU admission date |  |  |  |  | 0.21 |
|  | Before November 1, 2020 | 137 (42) | 91 (44) | 46 (37) |  |
|  | After November 1, 2020 | 191 (58) | 114 (56) | 77 (63) |  |
| Age, years |  | 72 (61, 79) | 73 (63, 80) | 70 (60, 78) | 0.056 |
| Female sex |  | 112 (34) | 64 (31) | 48 (39) | 0.15 |
| Body mass index, kg/m^2^ |  | 22.2 (20.1, 24.8) | 22.3 (20.1, 24.8) | 22.2 (20.1, 24.6) | 0.94 |
| ICU admission category |  |  |  |  | 0.35 |
|  | Non-scheduled surgery | 42 (13) | 29 (14) | 13 (11) |  |
|  | Medical | 286 (87) | 176 (86) | 110 (89) |  |
| Disease category |  |  |  |  | 0.016 |
|  | Cardiovascular disorder | 53 (16) | 37 (18) | 16 (13) |  |
|  | Respiratory disorder | 27 (8.2) | 21 (10) | 6 (4.9) |  |
|  | Gastrointestinal disorder | 41 (12) | 26 (13) | 15 (12) |  |
|  | Neurologic disorder | 107 (33) | 54 (26) | 53 (43) |  |
|  | Trauma | 58 (18) | 35 (17) | 23 (19) |  |
|  | Others | 42 (13) | 32 (16) | 10 (8.1) |  |
| Sepsis |  | 37 (11) | 27 (13) | 10 (8.1) | 0.16 |
| APACHE II score |  | 18 (14, 24) | 20 (15, 27) | 15 (12, 20) | <0.001 |
| SOFA score |  | 6 (4, 8) | 6 (4, 9) | 5 (3, 6) | <0.001 |
| Charlson comorbidity index score | | 1 (0, 3) | 1 (0, 3) | 1 (0, 2) | 0.001 |
| A1C, |  | 5.8 (5.5, 6.5) | 6.1 (5.6, 6.9) | 5.6 (5.3, 6.0) | <0.001 |
| ADAG ^a^, mg/dl |  | 120 (111, 140) | 128 (114, 151) | 114 (105, 126) | <0.001 |
| Diagnosed diabetes |  | 98 (30) | 83 (40) | 15 (12) | <0.001 |
| **Management in ICU** |  |  |  |  |  |
| Vasopressor use |  | 88 (27) | 63 (31) | 25 (20) | 0.039 |
| Mechanical ventilation |  | 153 (47) | 101 (49) | 52 (42) | 0.22 |
| Renal replacement therapy | | 22 (6.7%) | 15 (7.3) | 7 (5.7) | 0.57 |
| **Glycemic profile^b^** |  |  |  |  |  |
| The number of blood glucose measurements | | 14.0 (11.0, 17.0) | 15.0 (12.0, 19.0) | 13.0 (10.0, 15.0) | <0.001 |
| Mean blood glucose, mg/dl | | 130 (114, 158) | 149 (132, 183) | 110 (102, 118) | <0.001 |
| Coefficient of variation, % | | 19 (14, 27) | 23 (17, 31) | 14 (10, 18) | <0.001 |
| Hypoglycemia (< 70 mg/dL) | | 24 (7.3) | 16 (7.8) | 8 (6.5) | 0.66 |
| Moderate hypoglycemia (≥ 40mg/dl, < 70mg/dl) | | 20 (6.1) | 13 (6.3) | 7 (5.7) | 0.81 |
| Severe hypoglycemia (< 40mg/dl) | | 4 (1.2) | 3 (1.5) | 1 (0.8) | >0.99 |
| TIR of absolute normoglycemia^c^, % | | 70 (33, 91) | 47 (17, 67) | 94 (88, 100) | <0.001 |
| Time below 70 mg/dl, % | | 0.00 (0.00, 0.00) | 0.00 (0.00, 0.00) | 0.00 (0.00, 0.00) | 0.62 |
| Time above 140 mg/dl, % | | 29 (8, 63) | 50 (33, 81) | 0 (0, 11) | <0.001 |
| TIR of relative normoglycemia^d^, % | | 83 (65, 100) | 72 (58, 86) | 95 (88, 100) | <0.001 |
| TIR of relative normoglycemia^d^ > 80 % | | 172 (52) | 69 (34) | 103 (84) | <0.001 |
| Time below 70 % of ADAG^a^, % | | 0 (0, 9) | 0 (0, 12) | 0 (0, 6) | 0.34 |
| Time above 140 % of ADAG^a^, % | | 7 (0, 25) | 19 (0, 33) | 0 (0, 2) | <0.001 |
| **Outcomes** |  |  |  |  |  |
| ICU length of stay, days |  | 4.7 (2.8, 8.4) | 5.1 (2.9, 8.4) | 4.2 (2.7, 8.8) | 0.42 |
| ICU mortality |  | 20 (6.1) | 18 (8.8) | 2 (1.6) | 0.009 |
| Hospital length of stay, days | | 20 (13, 33) | 21 (13, 36) | 20 (12, 30) | 0.38 |
| hospital mortality |  | 35 (11) | 30 (15) | 5 (4.1) | 0.003 |

Data are expressed as median (interquartile range) or number (%)

*TIR* time in range, *APACHE* Acute Physiology and Chronic Health Evaluation, *SOFA* Sequential Organ Failure Assessment, *ADAG* A1C-derived average glucose

^a^ A1C-derived average glucose (mg/dl) can be obtained from the following formula: 28.7×A1C (%) -46.7

^b^ During the first 72 h after ICU admission or ICU stay, whichever shorter

^c^ Absolute normoglycemia was defined as measured blood glucose levels in the range of 70 to 140 mg/dl.

^d^ Relative normoglycemia was defined as measured blood glucose levels in the range of 70 to 140 of ADAG.

**Supplementary Table 5** The details of multivariate logistic regression analyses for mortality.

|  | Covariates | Adjusted OR (95% CI) | *P* value |
| --- | --- | --- | --- |
| Model 1 |  |  |  |
|  | Age, years | 0.98 (0.95,1.01) | 0.170 |
|  | Female sex | 1.48 (0.65,3.36) | 0.353 |
|  | APACHE II score, point | 1.12 (1.07,1.18) | < 0.001 |
|  | Charlson comorbidity index, point | 1.03 (0.86,1.24) | 0.719 |
|  | TIR of relative normoglycemia > 80% (vs. ≤ 80%) | 0.16 (0.06,0.43) | < 0.001 |
| Model 2 |  |  |  |
|  | Age, years | 0.99 (0.96,1.02) | 0.491 |
|  | Female sex | 1.43 (0.65,3.18) | 0.375 |
|  | APACHE II score, point | 1.13 (1.07,1.18) | < 0.001 |
|  | Charlson comorbidity index, point | 1.06 (0.88,1.26) | 0.551 |
|  | TIR of absolute normoglycemia > 80% (vs. ≤ 80%) | 0.44 (0.15,1.23) | 0.118 |

*APACHE II* Acute Physiology and Chronic Health Evaluation II*, TIR* time in range, *OR* odds ratio

**Supplementary Figure 2. Adjusted odds ratio of the time in range of absolute normoglycemia for in-hospital mortality according to the logistic regression model.**


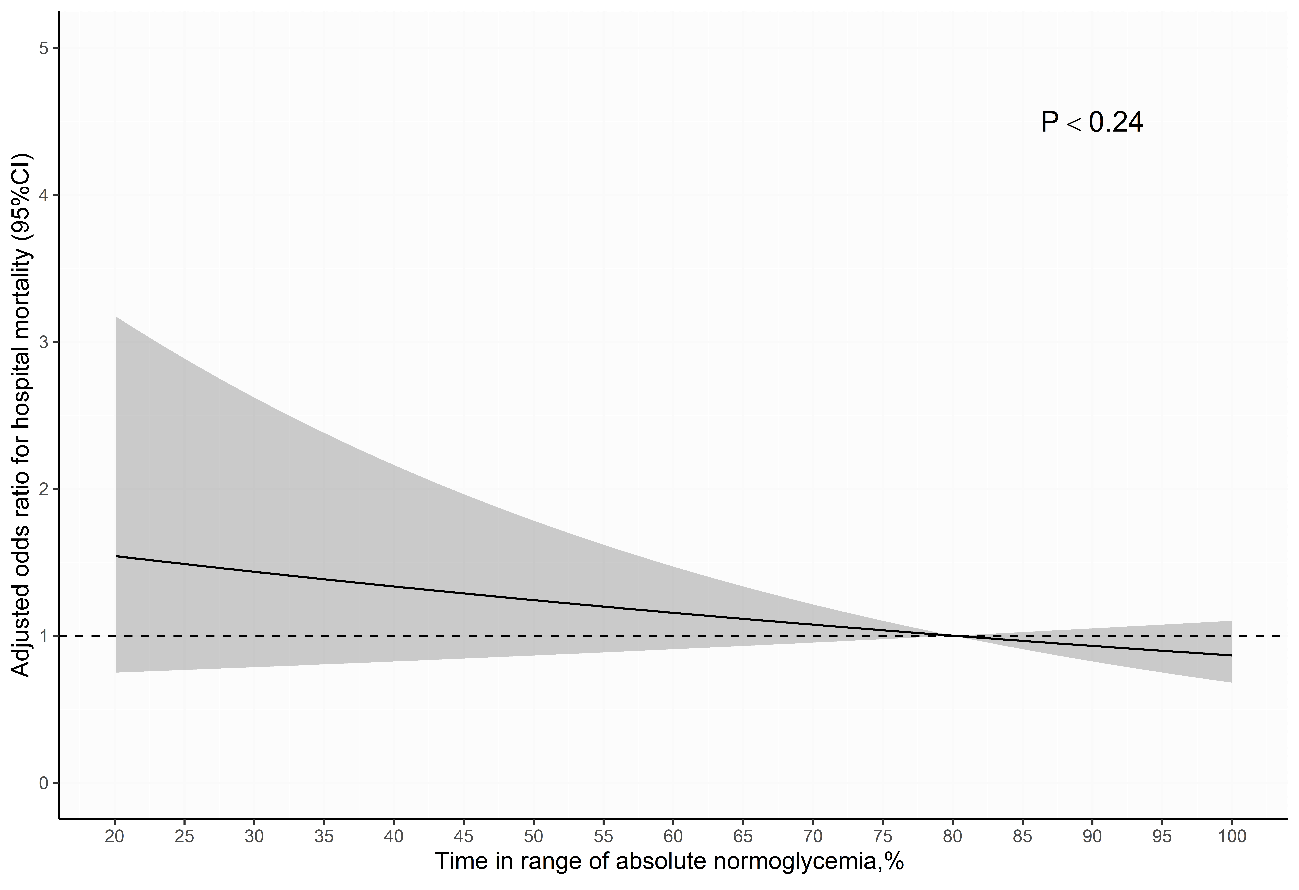


*CI* confidence interval

**Supplementary Table 6** Adjusted odds ratios of time in the range of absolute normoglycemia > 80% for in-hospital mortality according to A1C levels.

| Subgroup | TIR ≤ 80 % | TIR > 80 % |  | Adjusted OR (95 CI) | *P* value | *P* for interaction |
| --- | --- | --- | --- | --- | --- | --- |
|  | no. deaths/no. of patients (%) | |  |  |  |  |
| All | 30/205 (15) | 5/123 (4.1) |  | 0.44 (0.15,1.23) | 0.12 |  |
| A1C subgroup |  |  |  |  |  | 0.053 |
| < 6.5% | 24/130 (19) | 4/115 (3.5) |  | 0.28 (0.09,0.89) | 0.031 |  |
| ≥ 6.5% | 6/75 (8.0) | 1/8 (13) |  | 11.39 (0.59,221.21) | 0.11 |  |

*TIR* time in range, *OR* odds ratio, *CI* confidence interval

**Supplementary Table 7** Adjusted odds ratios of time in the range of relative normoglycemia > 80% for in-hospital mortality according to blood glucose measurement interval.

| Subgroup | TIR ≤ 80 % | TIR > 80 % |  | Adjusted OR (95 CI) | *P* value | *P* for interaction |
| --- | --- | --- | --- | --- | --- | --- |
|  | no. deaths/no. of patients (%) | |  |  |  |  |
| All | 30/205 (15) | 5/123 (4.1) |  | 0.44 (0.15, 1.23) | 0.12 |  |
| measurement interval |  |  |  |  |  | 0.81 |
| < 4.7 h | 22/98 (22) | 3/65 (4.6) |  | 0.17 (0.04, 0.55) | 0.008 |  |
| ≥ 4.7 h | 6/58 (10) | 1/25 (4.0) |  | 0.14 (0.02, 0.67) | 0.024 |  |

*TIR* time in range, *OR* odds ratio, *CI* confidence interval

**Supplementary Table 8** Time-varying Cox regression analysis for 28-day mortality

| Covariates | Adjusted hazard ratio (95% CI) | *P* value |
| --- | --- | --- |
| Age, years | 0.98 (0.96, 1.01) | 0.287 |
| Female sex | 1.22 (0.62, 2.41) | 0.571 |
| APACHE II score, point | 1.11 (1.07, 1.16) | < 0.001 |
| Charlson comorbidity index, point | 1.01 (0.85,1.20) | 0.913 |
| TIR of relative normoglycemia > 80% (vs. ≤ 80%) | 0.21 (0.08,0.58) | 0.002 |

*APACHE II* Acute Physiology and Chronic Health Evaluation II*, TIR* time in range, *CI* confidence interval
